# Characterization of the Geranylgeranyl Diphosphate Synthase Gene in *Acyrthosiphon pisum* (Hemiptera: Aphididae) and Its Association With Carotenoid Biosynthesis

**DOI:** 10.3389/fphys.2019.01398

**Published:** 2019-11-12

**Authors:** Bi-Yue Ding, Jinzhi Niu, Feng Shang, Li Yang, Teng-Yu Chang, Jin-Jun Wang

**Affiliations:** ^1^Key Laboratory of Entomology and Pest Control Engineering, College of Plant Protection, Southwest University, Chongqing, China; ^2^International Joint Laboratory of China-Belgium on Sustainable Crop Pest Control, State Cultivation Base of Crop Stress Biology for Southern Mountainous Land, Academy of Agricultural Sciences, Southwest University, Chongqing, China

**Keywords:** geranylgeranyl diphosphate synthase, carotenoid biosynthesis, horizontal gene transfer, RNAi, aphid

## Abstract

Carotenoids play many crucial roles in organisms. Recently, the *de novo* synthesis of carotenoids has been reported in pea aphid (*Acyrthosiphon pisum*) through horizontally transferred genes. However, their upstream pathway in the pea aphid is poorly understood. Geranylgeranyl diphosphate synthase (GGPPS) is the functional enzyme in the synthesis of geranylgeranyl diphosphate (GGPP) which is a precursor for the biosynthesis of many biological metabolites, including carotenoid synthesis. In this study, we performed a series of experiments to characterize *GGPPS* gene and its association with carotenoid biosynthesis. (1) determining the transcript abundance and carotenoid content in two geographical strain with red and green morphs, and (2) examining the abundance of carotenoid related genes and carotenoid levels after silencing of *GGPPS* in both red and green morphs. We observed that *GGPPS* was more highly expressed in the green morph than in the red morph of two strains of the pea aphid. The total level of carotenoids was also higher in green morphs than in red morphs in both strains. In addition to the total carotenoid difference, the carotenoids found in the two morphs also differed. There were α-carotene, β-carotene, and γ-carotene in the green morphs, but three additional carotenoids, including *cis*-torulene^∗^, *trans*-torulene^∗^, and 3,4-didehydrolycopene^∗^, were present in the red morphs. Silencing the *GGPPS* by RNAi in both the red and green morphs decreased the expression of some carotenoid biosynthesis-related genes, including carotenoid synthase/cyclase genes and carotenoid desaturase genes in green morphs. Carotenoid levels were decreased in both green and red morphs. However, the specific carotenoids present were not changed after silencing *GGPPS*. These results demonstrated that *GGPPS* may act as the upstream enzyme to influence the synthesis of the total amount of carotenoids. The present study provided important molecular evidence for the conserved roles of *GGPPS* associated with carotenoids biosynthesis and will enhance further investigation on the mechanisms of carotenoid biosynthesis in pea aphid.

## Introduction

Carotenoid is a generic term for a natural pigment, common in animals, higher plants, fungi, algae, and bacteria. Carotenoids play many crucial roles in organisms, including photosynthesis (Niedzwiedzki et al., 2017), protection of photo-oxidation (Frank and Brudvig, 2004), diapause (Bryon et al., 2017), and biological regulation (Heath et al., 2013). There are three ways that insects obtain carotenoids. These include dietary ingestion (Shimizu et al., 1981), endosymbionts (Sloan and Moran, 2012), and *de novo* synthesis. Synthesis has been reported in piercing-sucking pests, such as aphids, mosquitoes, and mites, through functional carotenoid biosynthetic genes, which were originally horizontally transferred from fungi (Moran and Jarvik, 2010; Altincicek et al., 2012; Cobbs et al., 2013). In the pea aphid, *Acyrthosiphon pisum*, carotenogenic genes are derived from fungi via horizontal gene transfer and through a series of duplication induced three Carotenoid Synthase or Cyclase genes (*CscA*-*C*) and four Carotenoid Desaturase genes (*CdeA*-*D*) (Moran and Jarvik, 2010; Supplementary Figure S1). In plants, bacteria, fungi, and algae, GGPP is synthesized by *GGPPS* and used as a precursor for the biosynthesis of carotenoids (Misawa et al., 1990; Hundle et al., 1994; Bartley and Scolnik, 1995; Mende et al., 1997; Cazzonelli and Pogson, 2010; Yang et al., 2016). However, the upstream pathway of *de novo* carotenoid synthesis in the pea aphid is poorly understood.

Geranylgeranyl diphosphate (GGPP) is a 20C organic compound synthesized by geranylgeranyl diphosphate synthase (GGPPS) through the head-to-tail condensation of three isopentenyl pyrophosphate (IPP) groups to the allyl head of dimethylallyl pyrophosphate (DMAPP). Some GGPPS can alternatively use geranyl pyrophosphate (GPP) or farnesyl pyrophosphate (FPP) as a substrate to produce GGPP (Sagami et al., 1993; Zhang and Li, 2014). Generally, GGPPS contains five highly conserved motifs, and the first and second aspartate-rich (FARM and SARM) motifs DDxx(xx)D are deemed to be the binding and catalysis sites in prenyltransferases (Quondam et al., 1997; Chang et al., 2006). According to the amino acid residues of the fourth and fifth positions before the first DDxx(xx)D, GGPPS is divided into three types: type-I (archaea), type II (plants and bacteria), and type III (yeasts and animals) (Barbar et al., 2013). The GGPPS in insects belong to type III GGPPS and they have an extra motif VI (GxNP) (Zhang and Li, 2014; Yang et al., 2016).

The number of *GGPPS* varies among species, and different *GGPPS* may produce GGPP using different metabolic pathways (Okada et al., 2000). For example, 12 *GGPPS* have been identified and 10 are functional that can synthesize GGPP from *Arabidopsis thaliana*. These genes are distributed in different subcellular compartments and display different expression patterns and most *GGPPSs* are distributed in plastids and expressed in the specific root or seed tissues (Lange and Ghassemian, 2003; Beck et al., 2013). However, *GGPPS11* (At4g36810) is widely expressed in photosynthetic tissues to provide GGPP for the biosynthesis of chlorophylls, carotenoids, or plastoquinones, which are vital for photosynthesis in *A. thaliana* (Okada et al., 2000; Beck et al., 2013; Coman et al., 2014). Two types of *GGPPS* (the first type is encoded GGPP for primary metabolism and the second one is responsible for secondary metabolism) have been isolated in *Gibberella fujikuroi* (Bettina, 2005; Singkaravanit et al., 2010). Only one *GGPPS* is found in most insects, such as the fruit fly *Drosophila melanogaster* (Lai et al., 1998), bumblebee, *Bombus terrestris* (Prchalová et al., 2016), spruce budworm, *Choristoneura fumiferana* (Barbar et al., 2013), and cotton aphid, *Aphis gossypii* (Zhang and Li, 2014).

Geranylgeranyl diphosphate synthase acts as a rate-limiting enzyme from IPP transformed to GGPP, and its enzyme activity not only affects the biosynthesis of carotenoids and chlorophylls, but it also affects the synthesis of other products derived from the isoprenoid pathway (Zhang and Li, 2014; Zhou et al., 2017). Targeting the isoprenoid pathway has been widely used in clinical agents (Swanson and Hohl, 2006) and the inhibitor of GGPPS could provide an alternative way in dealing with the diseases involving geranylgeranylation (Wiemer et al., 2011; Varney et al., 2018; Waller et al., 2019). In *D. melanogaster*, the geranylgeranyl pyrophosphate synthase-encoding gene, *quemao*, is critical in isoprenoids biosynthesis and heart development (Lai et al., 1998; Yi et al., 2006). GGPP synthesized by *GGPPS* is used to produce a diterpene in *Reticulitermes speratus* (Hojo et al., 2011). Silencing of *GGPPS* influences the biosynthesis of alarm pheromone (*E*)-β-farnesene (EβF) in *A. gossypii* (Sun and Li, 2018).

In this study, we report the identification of a single *GGPPS* in *A. pisum* and *GGPPS* expression and carotenoid content in two strains (NY and GS) of *A. pisum*. The expression patterns of *GGPPS*, carotenoid synthase/cyclase and desaturase genes, and carotenoid content were evaluated upon silencing of *GGPPs* in *A. pisum*. These results provide a new approach to the molecular regulation of carotenoid and promote the understanding of carotenoid biosynthesis in aphids.

## Materials and Methods

### Insects

To clarify the potential role of GGPPS in pea aphid, two population of *A. pisum* was used in the present study. The New York (NY) strain of *A. pisum* was originally collected from an alfalfa field in New York City, United States, in 2012. The Gansu (GS) strain was collected from an alfalfa field in Gansu Province, China, in 2016. These two strain kept in the lab for more than 3 years, without any exposure to pesticides and were fed separately on broad bean (*Vicia faba*) under 22 ± 1°C, 60 ± 10% relative humidity, and a 16:8 h (light: dark) photoperiod for more than 30 generations before applied in this study. All of the aphids were reared at a relatively low density (less than 30 individuals per seedling) in a continuous culture before being used in experiments. All of the progeny were produced by asexual reproduction.

### cDNA Synthesis and *GGPPS* Sequence Confirmation

Total RNAs used for *GGPPS* sequence confirmation and expression profiles were extracted using the TRIzol kit (Invitrogen, Carlsbad, CA, United States) according to the manufacturer’s instructions. DNase I (Promega, Madison, WI, United States) and PrimeScript RT Reagent Kit (Takara, Dalian, China) were used to removing possible genomic DNA contamination and synthesize the first-strand cDNA. The synthesized cDNA was stored at −20°C until use.

The *GGPPS* sequence was retrieved from both the genome database of *A. pisum* (AphidBase)^1^ and the NCBI database and the sequence was confirmed by sequencing the RT-PCR products by Sanger sequencing (Supplementary Table S1). Briefly, the PCR reactions were performed in a C1000^TM^ thermal cycler by an initial denaturation for 3 min at 95°C, followed by 35 cycles of 95°C for 30 s, 55°C to 60°C for 30 s, 72°C extension for 1 min, and a final extension for 10 min at 72°C. The reaction mixtures contained 10 × PCR buffer (Mg^2+^ free), 2.5 mM Mg^2+^, 2.5 mM dNTP mix, each specific primer (10 mM), and rTaq (Takara). The PCR products were purified and then ligated into a pGEM-T easy vector (Promega), which were then sequenced on an ABI Model 3100 automated sequencer (Invitrogen Life Technologies, Shanghai, China) to verify the *GGPPs* sequence.

### Phylogenetic Analysis

To illustrate the evolutionary positions of GGPPS in the pea aphid, the relative homologies from different representative classifications, including insect, vertebrate, yeast, bacterium, and plant were used to construct the phylogenetic tree. All of the sequences were obtained from NCBI^2^. The full-length amino acid sequences were aligned using MEGA 5.05 with the default settings in ClustalW with the maximum likelihood method. Bootstrap values were calculated based on 1000 replicates (Tamura et al., 2011). Sequence alignment of GGPPS with the pea aphid and several other species were produced with JalView 2.9 (Waterhouse et al., 2009).

### Spatiotemporal Expression and Sample Preparation

To detect the expression of *GGPPS* in two color types of an aphid, we collected red and green adults (within 12 h after the fourth instar nymph molt) from the NY and GS strains. In each group, 10 aphids were pooled as one biological replicate, and 4 biological replicates were included.

For determining the expression patterns of *GGPPS* at different developmental stages and tissues, we collected different developmental stages, namely, first, second, third, and fourth instar nymphs, and adults from the green morph of NY strain. For the nymphs, each stage was collected at three time points, namely, the early, middle, and late stages of each instar. In adults, four age time points were collected, namely, 8, 9, 12, and 15 days aphids (Supplementary Figure S2). Thirty insects were pooled as one sample. Tissues from the brain (200 individuals), stylet (200 individuals), integument (50 individuals), muscle (100 individuals), gut (100 individuals), fat body (100 individuals), and embryo (50 individuals) were excised from adults (within 12 h after the fourth instar nymph molt). Four biological replicates were performed for each group. All of the RNA samples were extracted by TRIzol and stored at −80°C until use.

### Quantitative Real-Time PCR (RT-qPCR)

RT-qPCR primers were designed using PRIMER 3.0^3^ (Supplementary Table S1). The RT-qPCR reaction mixtures contained nuclease-free water, each primer (0.2 mM), cDNA template, and NovoStart SYBR qPCR SuperMix (Novoprotein, Shanghai, China). The cDNA template used in the RT-qPCR reaction was diluted from the originally synthesized cDNA above with nuclease-free water to a final concentration of approximately 500 ng/μL. The reaction was performed on a Bio-Rad CFX Connect Real-Time System (Bio-Rad, Hercules, CA, United States) by following: 95°C for 120 s, 40 cycles of 95°C for 15 s, and 60°C for 30 s, and a final cycle of 60°C for 5 s and melting curve data were collected every 0.5°C until 95°C. A standard curve was established for each primer pair to determine the amplification efficiency. The reference genes *elongation factor-1 alpha* (*EF1*α) (GenBank accession number: AY219737.1) and *ribosomal protein S20* (*RPS20*) (GenBank accession number: NM_001162819.2) were used to normalize the gene expression levels (Chen et al., 2016). The relative gene expression levels, based on two reference genes, were calculated using qBASE (Hellemans et al., 2007).

### Carotenoid Extraction and HPLC Analysis

In parallel with part 2.4, we also collected red and green newly emerged adults within 12 h for determining the carotenoid content from the NY and GS strains. Twenty adults were pooled as a biological replicate, and four biological replicates were included in each group.

Carotenoids were extracted by following a standard protocol with slight modifications (Valmalette et al., 2012). Briefly, aphids were first ground in 200 μL of extraction solvent (acetone/hexane, 1:1 v/v, containing 0.1% of BHT as an antioxidant). Then, the extraction solvent was added to 1 mL and vortexed for 30 min. The extracts were centrifuged at 12,000 × *g* at 4°C for 10 min. The organic phases were dried under anhydrous sodium sulfate, filtered, and concentrated to dryness with a vacuum concentrator. The residue was dissolved in 50 μL methyl tert-butyl ether (MTBE) (containing 0.1% of BHT as an antioxidant). Samples were placed in amber vials before high-performance liquid chromatography (HPLC) analysis.

Carotenoids were separated along a C30 column (250 × 4.6 mm, 5 μm particle size) (YMC Co., Ltd., Kyoto, Japan) (Zhang et al., 2009; Valmalette et al., 2012). The mobile phases were acetonitrile/methanol (3:1 v/v, containing 0.1% of BHT as antioxidant) as eluent A, and MTBE (containing 0.1% of BHT as antioxidant) as eluent B. Flow rate was fixed at 1 mL/min and the column temperature was set at 30°C. A gradient program was performed: 0–10 min, 95% A/5% B, isocratic elution: 10–19 min, 86% A/14% B; 19–29 min, 75% A/25% B; 29–54 min, 50% A/50% B; 54–66 min, 26% A/74% B; 67–67 min, back to the initial conditions for re-equilibration. The injection volume was 20 μL and the detection was monitored at 450 nm. The α-carotene, β-carotene, and γ-carotene were identified and quantitatively determined by the standards using Agilent 1260 LC (Agilent Technologies, Santa Clara, CA, United States). The *cis*-torulene^∗^, all *trans*-torulene^∗^, and 3, 4-didehydrolycopene^∗^ were identified according to previous studies and calculated based on the β-carotene equivalent (Tsuchida et al., 2010; Valmalette et al., 2012).

Serial dilutions of carotenoid standards were used to establish the standard curves. For α-carotene standard (Sigma, St. Louis, MO, United States), 4, 2, 1, 0.8, 0.6, and 0.4 μg/mL were included. For β-carotene standard (Sigma), 4, 2, 1, 0.8, and 0.6 μg/mL were included and for γ-carotene standard (Sigma), concentrations were 1, 0.8, 0.6, 0.4, and 0.2 μg/mL. The calibration curve and coefficients were listed in Supplementary Figures S3–S5.

### RNAi Bioassays

The double-strand RNA (dsRNA) was synthesized using a Transcript Aid T7 High Yield Transcription Kit (Thermo Scientific, Wilmington, DE, United States) according to the manufacturer’s instructions (Supplementary Table S1). The dsRNA was diluted with Nuclease-free water to a final concentration of 5,000 ng/μL.

Newly emerged green and red form adults (≤12 h old) from the NY strain were used in the RNAi experiments. The dsRNA injection was accomplished as in a previous study (Ye et al., 2019). Briefly, 600 ng of ds*GGPPS* and ds*GFP* (negative control) were injected into the aphids using an M3301 micromanipulator (World Precision Instruments, Sarasota, FL, United States). In each treatment, 25 aphids were injected as one biological replicate and 4 biological replicates were completed. After injection, the aphids were moved to broad bean leaves. After 36 h, in each biological replicate, 5 aphids were collected to detect gene expression levels and 20 aphids were collected for analyzing carotenoids content.

### Statistical Analysis

Significant differences in carotenoid content in red and green morphs from the two strains and transcript levels of *GGPPS* in tissues were tested by one-way analysis of variance (ANOVA) followed by Tukey’s honestly significant difference test (Tukey’s HSD) for multiple samples comparison (*P* < 0.05). Significant differences in transcript levels of *GGPPS* in red and green morphs from the two strains were tested by Student’s *t*-test. Student’s *t*-tests were used to analyze the significance of gene expression level and carotenoid content between the ds*GGPPS* treatment and control (ds*GFP*). All of the statistical analyses were performed using SPSS version 20.0 software (IBM, Armonk, NY, United States).

## Results

### Phylogenetic Analysis of GGPPS

**FIGURE 1:**
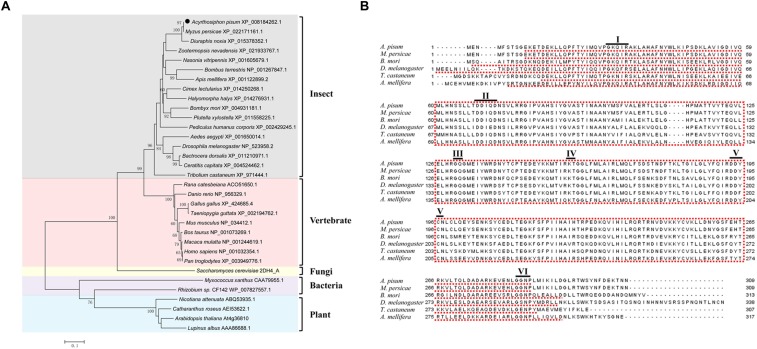
Phylogenetic tree and sequence alignments of GGPPS. **(A)** Phylogenetic tree of GGPPS generated by MEGA 5.05. **(B)** Alignment of the amino acid sequence of *Acyrthosiphon pisum* GGPPS with other insect GGPPS. *A. pisum*, *Acyrthosiphon pisum*; *M. persicae*, *Myzus persicae*; *B. mori*, *Bombyx mori*; *D. melanogaster*, *Drosophila melanogaster*; *T. castaneum*, *Tribolium castaneum*; *A. mellifera*, *Apis mellifera*. Conserved motifs are underlined, and the first and second aspartate-rich motifs (FARM: motif II and SARM: motif V, respectively) are DDIQD and DDYCN.

The open reading frame of *GGPPS* in *A. pisum* contains 930 bp and encodes 309 amino acid residues. The predicted isoelectric point of GGPPS is 6.32 and the molecular weight is 35 kDa. To investigate the evolutionary relationships of the GGPPS gene among different organisms, the full-length amino acid of GGPPS from seven insects, nine vertebrates, one fungus, two bacteria, and four plants were used to construct a phylogenetic tree. The GGPPS of insects and vertebrates belong to a large branch, close to fungal GGPPS (*Saccharomyces cerevisiae*) with a high value. GGPPS from *A. pisum* was the closest to the other two aphid species, *Myzus persicae* and *Diuraphis noxia*. GGPPS from bacteria and plants were clustered into another branch (Figure 1A).

Sequence analysis showed that GGPPS from *A. pisum* and other insects were conserved and mainly include six conserved motifs: motif I (GKxxR), motif II (DDIQ/ED), motif III (GQ), motif IV (KT), motif V (DDYCN), and motif VI (GxNP). The first aspartate-rich motif (FARM) of *A. pisum* GGPPS was DDIQD, while in the second aspartate-rich motif (SARM), the last residue was an asparagine (DDYCN) instead of aspartate (Figure 1B and Supplementary Figure S6).

### Expression Patterns of *GGPPS* in Green and Red Morphs

**FIGURE 2:**
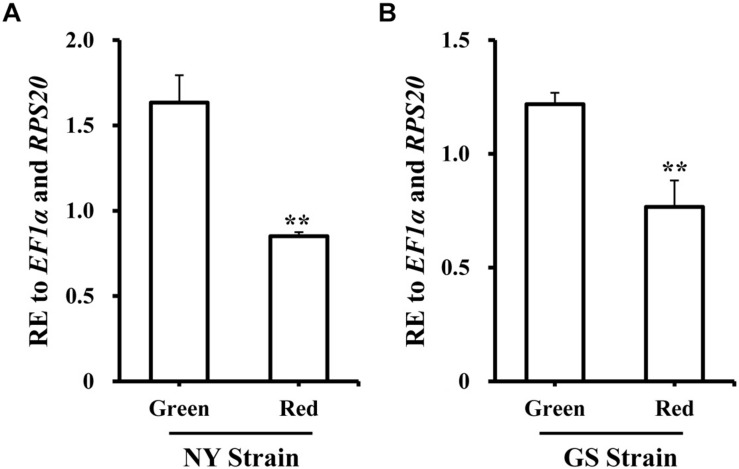
Expression profiles of *GGPPS* in green and red morphs of the NY and GS strains of *Acyrthosiphon pisum*. **(A)** Expression patterns of *GGPPS* in red and green morphs of NY strain. **(B)** Expression patterns of *GGPPS* in red and green morphs of GS strain. A significant difference between red and green morphs is indicated by asterisks (^∗∗^*P* < 0.01).

To better improve our understanding of the role in pea aphid, the expression level of *GGPPS* was analyzed in spatiotemporal dynamics and different body-color. Firstly, samples from the different developmental stages and tissues from the NY strain green morph were collected. *GGPPS* was consistently expressed during all of the tested stages and tissues. From the 1st to the 3rd instar nymphs, *GGPPS* expression slightly decreased and then increased along with aphid development (Supplementary Figure S7A). *GGPPS* were significantly higher in the brain and embryo tissues compared to other tissues, including stylet, integument, muscle, gut, and fat body (Supplementary Figure S7B). Secondly, the expression patterns of *GGPPS* were determined in red and green morphs of *A. pisum* from the NY and GS strains. In both strains, *GGPPS* was more highly expressed in the green morph compared to the red morph (Figure 2).

### Carotenoid Contents in Green and Red Morphs

Next, we determined the carotenoid contents of red and green morphs. In green morphs, only α-carotene, β-carotene, and γ-carotene were detected (Table 1). In red morphs, three additional carotenoids were detected, including *cis*-torulene^∗^, all *trans*-torulene^∗^, and 3,4-didehydrolycopene^∗^ (Table 1). The content of α-carotene, β-carotene, and total carotenoids was higher in the green morph compared to red morph in the NY and GS strains. No differences in compositions of individual carotenoids and the total content were observed between morphs of the NY and GS strains with the same body color (Table 1).

**TABLE 1:** Carotenoid content of green and red morphs from NY and GS strains of *Acyrthosiphon pisum*.

	**NY strain**		**GS strain**	
		
	**Green**	**Red**	**Green**	**Red**
α-carotene	0.4920 ± 0.0202 b	0.1846 ± 0.0123 a	0.4592 ± 0.0273 b	0.1798 ± 0.0147 a
β-carotene	3.9132 ± 0.1498 b	0.7828 ± 0.1569 a	3.7664 ± 0.2266 b	0.6316 ± 0.0831 a
γ-carotene	0.1066 ± 0.0102	0.1150 ± 0.0343	0.0737 ± 0.0115	0.0980 ± 0.0140
*cis*-torulene^∗^	ND a	0.8189 ± 0.2115 b	ND a	0.7188 ± 0.0796 b
All-*trans*-torulene^∗^	ND a	0.5111 ± 0.0925 b	ND a	0.4781 ± 0.0292 b
3, 4-didehydrolycopene^∗^	ND a	0.3595 ± 0.0642 b	ND a	0.3308 ± 0.0145 b
total	4.5118 ± 0.1776 b	2.7718 ± 0.5618 a	4.2993 ± 0.2630 b	2.4371 ± 0.2241 a

*^∗^ represents that the carotenoids were identified according to previous studies and calculated based on the β-carotene equivalent (Tsuchida et al., 2010; Valmalette et al., 2012). ND represents not detected. Lowercase letters (a and b) indicate significant differences in the content of the same carotenoid composition among different morphs or strains were tested by one-way ANOVA followed with Tukey’s honestly significant difference test for multiple samples comparison (*P* < 0.05).*

### Expression Profile of Carotenoids Associated Genes and Carotenoid Content Upon Silencing of *GGPPS*

**FIGURE 3:**
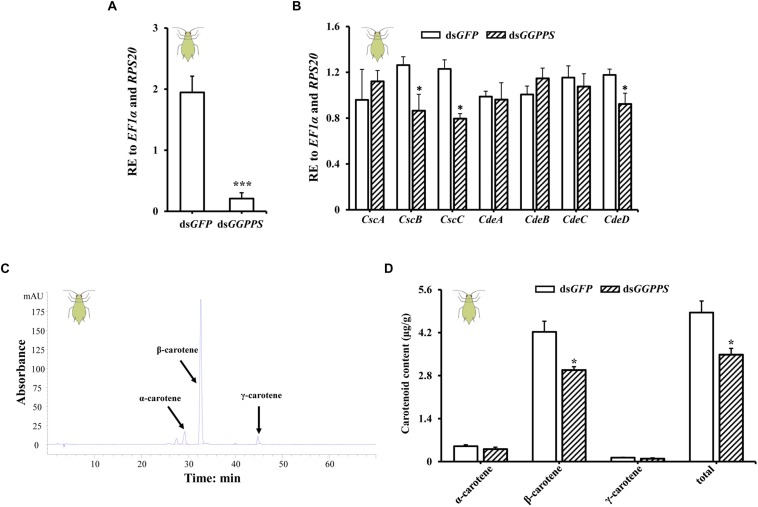
Expression patterns of *GGPPS*, carotenoid biosynthesis-related genes, and carotenoids content upon silencing of *GGPPS* in the green morph of *Acyrthosiphon pisum*. **(A)** RNAi efficiency of *GGPPS*. **(B)** Expression patterns of carotenoid biosynthesis-related genes after RNAi of *GGPPS*. **(C)** The prominent peak retention time of green morph by HPLC. Black arrows represent different carotenoids. **(D)** Carotenoid content of the green morph after silencing of *GGPPS*. Significant differences between ds*GFP* and ds*GGPPS* are indicated by asterisks (^∗^*P* < 0.05; ^∗∗∗^*P* < 0.001).

To future study the function of *GGPPS* in association with carotenoid biosynthesis, we examined the expression patterns of carotenoid biosynthesis-associated genes (including carotenoid synthase/cyclase genes and carotenoid desaturase genes obtained from fungi through horizontal gene transfer) and the carotenoid content in green and red morphs after RNAi of *GGPPS*. In green morphs, the expression level of *GGPPS* was significantly reduced by 89.4% after ds*GGPPS* injection (Figure 3A), and this decreased the expression levels of two carotenoid synthase/cyclase genes (*CscB* and *CscC*) and one carotenoid desaturase gene (*CdeD*). No significant effects on other carotenoid biosynthesis-related genes were observed (Figure 3B). The total carotenoid content was significantly decreased after silencing of *GGPPS* and the content of β-carotene was significantly reduced. Levels of α-carotene and γ-carotene were not significantly influenced and only slightly decreased (Figures 3C,D).

**FIGURE 4:**
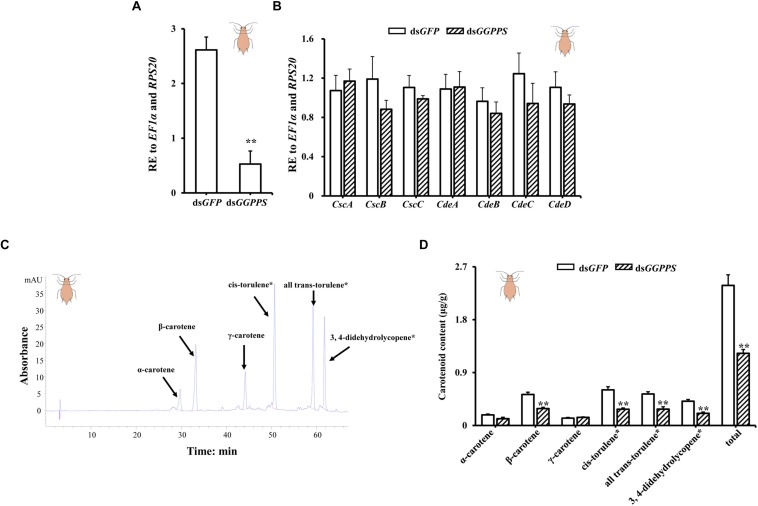
Expression patterns of *GGPPS*, carotenoid biosynthesis-related genes, and carotenoids content upon silencing of *GGPPS* in the red morph of *Acyrthosiphon pisum*. **(A)** RNAi efficiency of *GGPPS*. **(B)** Expression patterns of carotenoid biosynthesis-related genes after RNAi of *GGPPS*. **(C)** The prominent peak retention time of red morph by HPLC. Black arrows represent different carotenoids. **(D)** Carotenoid content of the red morph after silencing of *GGPPS*. Significant differences between ds*GFP* and ds*GGPPS* are indicated by asterisks (^∗∗^*P* < 0.01).

In the red morph, silencing produced an 80% reduction of *GGPPS* (Figure 4A), but there were no significant differences in the carotenoid biosynthesis-related genes. However, the expression of two carotenoid synthase/cyclase genes (*CscB* and *CscC*) and three carotenoid desaturase genes (*CdeB*, *CdeC*, and *CdeD*) decreased (Figure 4B). Similarly, the total carotenoid content was significantly reduced. Contents of β-carotene, *cis*-torulene^∗^, all *trans*-torulene^∗^, and 3,4-didehydrolycopene^∗^ were all significantly decreased, and α-carotene content decreased but there was no obvious effect on the content of γ-carotene (Figures 4C,D).

## Discussion

Geranylgeranyl diphosphate is a precursor for the biosynthesis of carotenoids and has been studied in organisms that synthesize carotenoids. Some insect species, such as aphids, can synthesize carotenoids via carotenoid biosynthesis-related genes obtained from fungi through horizontal gene transfer (Moran and Jarvik, 2010; Nováková and Moran, 2012). However, whether *GGPPS* is responsible for carotenoids biosynthesis in *A. pisum*, similar to reports in other organisms, has not been determined.

Based on the genome dataset of *A. pisum*, a single *GGPPS* in the isoprenoid biosynthesis pathway was identified. Sequence alignments of GGPPS with different insects showed that GGPPS are highly conserved, and the first five conserved motifs occurred in all *trans*-prenyltransferases. These results implied that GGPPS perform conserved functions in insects (Zhang and Li, 2014). GGPPS among animals (such as insects and vertebrates) and fungi group together and are referred to as type III GGPPS. Since fungi were the donors of the aphids horizontally transferred carotenoid-associated genes, it is reasonable to predict that GGPPS is the upstream enzyme determining the synthesis of aphid carotenoids. In addition, GGPPS could be categorized into three groups based on amino acid residues at the fourth or fifth location before the FARM or the insertion within the FARM. The pea aphid GGPPS was similar to type III GGPPS (Barbar et al., 2013), which is preferred for accepting FPP, compared to DMAPP, as an allylic substrate (Yang et al., 2014).

In plants, *GGPPS* are a small gene family and all homologs have tissue- and organelle-specific expression. *GGPPS4* (At2g23800) is highly expressed in flower, *GGPPS6* (At3g14530) is highly expressed in root and siliques, while *GGPPS11* (At4g36810) is presented high abundance in all organs and tissues and is the one mainly responsible for most metabolites in *A. thaliana* (Beck et al., 2013). However, we identified only one ortholog of *GGPPS* in pea aphid and the transcript was more abundant in the green morph than in the red morph. This indicates the association of GGPPS and carotenoid synthesis. Bioinformatics analysis has revealed the carotenoid-associated genes in the pea aphid genome (Moran and Jarvik, 2010; Nováková and Moran, 2012). However, the biological and ecological functions of these genes are unclear. In addition, the original horizontal gene transfer has also undergone a series of duplications and there are now seven carotenoid associated genes in pea aphids (Moran and Jarvik, 2010). This suggests that these genes have provided a selective advantage to aphids possessing them. Similarly, sap-feeding spider mites, such as *Tetranychus urticae* and *Panonychus citri*, obtained carotenoid biosynthesis-related genes through horizontal gene transfer, and one carotenoid desaturase gene is involved in carotenoid accumulation and diapause (Altincicek et al., 2012; Bryon et al., 2017). Upon silencing of *GGPPS* by RNAi, the expression level of some carotenoid biosynthesis-related genes was decreased, indicating these horizontally transferred genes are functional and linked with *GGPPS* in the pea aphid. In *A. thaliana*, a *GGPPS11* (At4g36810) knockdown strain (*ggpps11-5*) showed significantly reduced carotenoid levels compared to wild-type, while the lines of overexpression of *GGPS11* alone do not accumulate higher amounts of carotenoids (Ruiz-Sola et al., 2016; Camagna et al., 2019). In *Xanthophyllomyces dendrorhous*, an increase in the levels of *GGPPS* resulted in higher carotenoid content (Breitenbach et al., 2011). Red and green morphs of *A. pisum* were significantly different in their relative expression levels of *GGPPS* and this may be associated with the total levels of carotenoids in the two-color forms. Silencing of *GGPPS* decreased the carotenoid content rather than changing the carotenoid composition. These data are consistent with the finding that carotenoid content and compositions are different in the two-color morphs (green morphs contain higher carotenoids content than that in red morphs, while red contains a number of different carotenoids than that in green morphs) (Moran and Jarvik, 2010; Tsuchida et al., 2010). The body-color polyphenism (red and green) may be related to carotenoids composition rather than the level of total carotenoids content (Moran and Jarvik, 2010; Tsuchida, 2016). The differences of total carotenoids in the two-color forms of *A. pisum* are probably associated with biological and ecological adaptations, such as host location, energy reserves, and probing behavior (Tsuchida, 2016; Zhang et al., 2016), which will require further study.

Besides contributing to carotenoids biosynthesis, *GGPPS* may have multiple physiological functions. Silencing of *GGPPS* increased the EβF amount but didn’t change the body length, body width, emitting cornicle droplets, fecundity, and survival in *A. gossypii* (Sun and Li, 2018). The duplication of the *GGPPSs* might be involved in the evolution of the chemical defense in *Nasutitermes takasagoensis* (Hojo et al., 2019). The *GGPPS* (*qm*) mutant in *Drosophila* showed a “broken-hearted” phenotype in embryos (Yi et al., 2006).

In total, our results indicate there might be an association with higher expression of *GGPPS* in the green morph and a relatively higher carotenoid content. Although direct silencing of *GGPPS* decreased the carotenoid contents, this suggests that GGPP may act as the substrate for carotenoid synthesis by downstream horizontally transferred genes regardless of red or green aphids. Does this mean carotenoids synthesis is a case of the substrate (GGPP) the concentration-dependent manner in green aphids or *GGPPS* plays a regulatory role in carotenoids synthesis? Besides, in this study, we focused on the very upstream gene *GGPPS* (non-horizontal transferred gene) in the evaluation of its involvement of carotenoids biosynthesis, and further association with the seven horizontally transferred carotenoids associated genes (carotenoid synthase/cyclase genes and carotenoid desaturase genes). It still requires intensive studies on further investigation of these seven genes in involvement with host adaptation, specifically, why aphids need duplicated carotenoids genes (Moran and Jarvik, 2010; Nováková and Moran, 2012)? Which are absent in most insects. This provides a nice model to study how horizontal transferred genes in facilitating the better adaptation of insects. So far, this is not yet clear any aphid species that are lacking these horizontally transferred genes, thus it is not possible to perform a comparative study between aphids with carotenoids synthesis genes and aphids without carotenoids synthesis genes. Further studies may be helpful in using tools such as genome editing approach to analyze the association between *GGPPS* and carotenoids synthesis in aphids (Le Trionnaire et al., 2019).

## Data Availability Statement

All datasets generated for this study are included in the article/Supplementary Material.

## Ethics Statement

The research project was conducted on invertebrate species that are not subject to specific ethical issues and legislation.

## Author Contributions

B-YD, JN, and J-JW conceived and designed the experiments. B-YD performed all of the experiments with the help of FS, LY, and T-YC. JN and J-JW provided the materials. B-YD, JN, and FS analyzed data. B-YD, JN, FS, and J-JW wrote the manuscript.

## Conflict of Interest

The authors declare that the research was conducted in the absence of any commercial or financial relationships that could be construed as a potential conflict of interest.

## Footnotes

1 http://bipaa.genouest.org/is/aphidbase/

2 https://www.ncbi.nlm.nih.gov/

3 http://bioinfo.ut.ee/primer3-0.4.0/

## Supplementary Material

FIGURE S1 The suppositional carotenoid biosynthesis pathway in the pea aphid. [According to Moran and Jarvik (2010)].

FIGURE S2 Schematic diagram of samples collected for different developmental stages. N1-1: Newly enclosed nymph in 4 h; N1-2: middle of 1st instar nymph; N1-3: late stage of 1st instar nymph; N2-1: early stage of 2nd instar nymph; N2-2: middle stage of 2nd instar nymph; N2-3: late stage of 2nd instar nymph; N3-1: early stage of 3rd instar nymph; N3-2: middle stage of 3rd instar nymph; N3-3: late stage of 3rd instar nymph; N4-1: early stage of 4th instar nymph; N4-2: middle stage of 4th instar nymph; N4-3: late stage of 4th instar nymph; AD-1: 8 days old adult; AD-2: 9 days old; AD-3: 12 days old; AD-4: 15 days old.

FIGURE S3 Standard curve of α-carotene obtained by HPLC. The linear regression equation was generated using 0.08, 0.04, 0.02, 0.016, 0.012, and 0.008 μg concentrations.

FIGURE S4 Standard curve of β-carotene obtained by HPLC. The linear regression equation was generated using 0.08, 0.04, 0.02, 0.016, and 0.012 μg concentrations.

FIGURE S5 Standard curve of γ-carotene obtained by HPLC. The linear regression equation was generated using 0.02, 0.016, 0.012, 0.008, and 0.004 μg concentrations.

FIGURE S6 Alignment of the amino acid sequence of *Acyrthosiphon pisum* GGPPS with GGPPS from other species. Conserved motifs are indicated by the red box with dotted lines.

FIGURE S7 Expression patterns of *GGPPS* during different developmental stages and tissues in the green morph of *Acyrthosiphon pisum, NY strain*. **(A)** The expression patterns of *GGPPS* in different developmental stages. N1: 1st instar nymph, N2: 2nd instar nymph, N3: 3rd instar nymph, N4: 4th instar nymph, AD: adult. **(B)** Expression patterns of *GGPPS* in tissues. BR, brain; ST, stylet; IN, integument; MU, muscle; GU, gut; FB, fat body; EM, embryo. Mean (±SE) expression level was based on four biological replicates. Lowercase letters (a and b) above each bar indicate significant differences among tissues (One-way ANOVA followed by Tukey’s honestly significant difference multiple comparison test; *P* < 0.05).

TABLE S1 Primer sequences used for sequence confirmation, quantitative real-time PCR (qRT-PCR) and double stranded RNA (dsRNA) synthesis.

## References

[B1] Altincicek B. Kovacs J. L. Gerardo N. M. (2012). Horizontally transferred fungal carotenoid genes in the two-spotted spider mite *Tetranychus urticae*. *Biol. Lett.* 8 253–257. 10.1098/rsbl.2011.0704 21920958 PMC3297373

[B2] Barbar A. Couture M. Sen S. E. Béliveau C. Nisole A. Bipfubusa M. (2013). Cloning, expression and characterization of an insect geranylgeranyl diphosphate synthase from *Choristoneura fumiferana*. *Insect. Biochem. Mol. Biol.* 43 947–958. 10.1016/j.ibmb.2013.07.004 23907071

[B3] Bartley G. E. Scolnik P. A. (1995). Plant carotenoids: pigments for photoprotection, visual attraction, and human health. *Plant Cell* 7 1027–1038. 10.1105/tpc.7.7.1027 7640523 PMC160905

[B4] Beck G. Coman D. Herren E. Ruiz-Sola M. A. Rodríguez-Concepción M. Gruissem W. (2013). Characterization of the synthase gene family in *Arabidopsis thaliana*. *Plant Mol. Biol.* 82 393–416. 10.1007/s11103-013-0070-z 23729351

[B5] Bettina T. (2005). Gibberellin biosynthesis in fungi: genes, enzymes, evolution, and impact on biotechnology. *Appl. Microbiol. Biotechnol.* 66 597–611. 10.1007/s00253-004-1805-1 15578178

[B6] Breitenbach J. Visser H. Verdoes J. C. Ooyen A. J. J. V. Sandmann G. (2011). Engineering of geranylgeranyl pyrophosphate synthase levels and physiological conditions for enhanced carotenoid and astaxanthin synthesis in xanthophyllomyces dendrorhous. *Biotechnol. Lett.* 33 755–761. 10.1007/s10529-010-0495-2 21165672

[B7] Bryon A. Kurlovs A. H. Dermauw W. Greenha R. Riga M. Grbic M. (2017). Disruption of a horizontally transferred phytoene desaturase abolishes carotenoid accumulation and diapause in *Tetranychus urticae*. *Proc. Natl. Acad. Sci. U.S.A.* 114:E5871. 10.1073/pnas.1706865114 28674017 PMC5530703

[B8] Camagna M. Grundmann A. Bär C. Koschmieder J. Beyer P. Welsch R. (2019). Enzyme fusion removes competition for geranylgeranyl diphosphate in carotenogenesis. *Plant Physiol.* 179 1013–1027. 10.1104/pp.18.01026 30309967 PMC6393812

[B9] Cazzonelli C. I. Pogson B. J. (2010). Source to sink: regulation of carotenoid biosynthesis in plants. *Trends Plant Sci.* 15 266–274. 10.1016/j.tplants.2010.02.003 20303820

[B10] Chang T. H. Guo R. T. Ko T. P. Wang A. H. J. Liang P. H. (2006). Crystal structure of type-III geranylgeranyl pyrophosphate synthase from *Saccharomyces cerevisiae* and the mechanism of product chain length determination. *J. Biol. Chem.* 281 14991–15000. 10.1074/jbc.m512886200 16554305

[B11] Chen N. Fan Y. L. Bai Y. Li X. D. Zhang Z. F. Liu T. X. (2016). Cytochrome P450 gene, CYP4G51, modulates hydrocarbon production in the pea aphid, *Acyrthosiphon pisum*. *Insect Biochem. Mol. Biol.* 76 84–94. 10.1016/j.ibmb.2016.07.006 27425674

[B12] Cobbs C. Heath J. Stireman J. O. Abbot P. (2013). Carotenoids in unexpected places: gall midges, lateral gene transfer, and carotenoid biosynthesis in animals. *Mol. Phylogenet. Evol.* 68 221–228. 10.1016/j.ympev.2013.03.012 23542649

[B13] Coman D. Altenhoff A. Zoller S. Gruissem W. Vranová E. (2014). Distinct evolutionary strategies in the GGPPS family from plants. *Front. Plant Sci.* 5:230. 10.3389/fpls.2014.00230 24904625 PMC4034038

[B14] Frank H. A. Brudvig G. W. (2004). Redox functions of carotenoids in photosynthesis. *Biochemistry* 43 8607–8615. 10.1021/bi0492096 15236568

[B15] Heath J. J. Cipollini D. F. Stireman J. O. III (2013). The role of carotenoids and their derivatives in mediating interactions between insects and their environment. *Arthropod Plant Interact.* 7 1–20. 10.1007/s11829-012-9239-7

[B16] Hellemans J. Mortier G. Paepe A. D. Speleman F. Vandesompele J. (2007). qBase relative quantification framework and software for management and automated analysis of real-time quantitative PCR data. *Genome Biol.* 8:R19. 17291332 10.1186/gb-2007-8-2-r19 PMC1852402

[B17] Hojo M. Shigenobu S. Maekawa K. Miura T. Tokuda G. (2019). Duplication and soldier-specific expression of geranylgeranyl diphosphate synthase genes in a nasute termite *Nasutitermes takasagoensis*. *Insect Biochem. Mol. Biol.* 111:103177. 10.1016/j.ibmb.2019.103177 31228516

[B18] Hojo M. Toga K. Dai W. Yamamoto T. Maekawa K. (2011). High-level expression of the geranylgeranyl diphosphate synthase gene in the frontal gland of soldiers in *Reticulitermes speratus* (Isoptera: Rhinotermitidae). *Arch. Insect Biochem. Physiol.* 77 17–31. 10.1002/arch.20415 21308763

[B19] Hundle B. Alberti M. Nievelstein V. Beyer P. Kleinig H. Armstrong G. A. (1994). Functional assignment of *Erwinia herbicola* Eho10 carotenoid genes expressed in *Escherichia coli*. *Mol. Gen. Genet.* 245 406–416. 10.1007/bf00302252 7808389

[B20] Lai C. Mcmahon R. Young C. Mackay T. F. Langley C. H. (1998). quemao, a *Drosophila* bristle locus, encodes geranylgeranyl pyrophosphate synthase. *Genetics* 149 1051–1061. 9611212 10.1093/genetics/149.2.1051 PMC1460199

[B21] Lange B. M. Ghassemian M. (2003). Genome organization in *Arabidopsis thaliana*: a survey for genes involved in isoprenoid and chlorophyll metabolism. *Plant Mol. Biol.* 51 925–948. 12777052 10.1023/a:1023005504702

[B22] Le Trionnaire G. Tanguy S. Hudaverdian S. Gleonnec F. Richard G. Cayrol B. (2019). An integrated protocol for targeted mutagenesis with CRISPR-Cas9 system in the pea aphid. *Insect Biochem. Mol. Biol.* 110 34–44. 10.1016/j.ibmb.2019.04.016 31015023

[B23] Mende K. Homann V. Tudzynski B. (1997). The geranylgeranyl diphosphate synthase gene of *Gibberella fujikuroi*: isolation and expression. *Mol. Gen. Genet.* 255 96–105. 10.1007/s004380050477 9230902

[B24] Misawa N. Nakagawa M. Kobayashi K. Yamano S. Izawa Y. Nakamura K. (1990). Elucidation of the *Erwinia uredovora* carotenoid biosynthetic pathway by functional analysis of gene products expressed in *Escherichia coli*. *J. Bacteriol.* 172 6704–6712. 10.1128/jb.172.12.6704-6712.1990 2254247 PMC210783

[B25] Moran N. A. Jarvik T. (2010). Lateral transfer of genes from fungi underlies carotenoid production in aphids. *Science* 328 624–627. 10.1126/science.1187113 20431015

[B26] Niedzwiedzki D. M. Dilbeck P. L. Tang Q. Martin E. C. Bocian D. F. Hunter C. N. (2017). New insights into the photochemistry of carotenoid spheroidenone in light-harvesting complex 2 from the purple bacterium *Rhodobacter sphaeroides*. *Photosynth. Res.* 131 291–304. 10.1007/s11120-016-0322-2 27854005 PMC5313593

[B27] Nováková E. Moran N. A. (2012). Diversification of genes for carotenoid biosynthesis in aphids following an ancient transfer from a fungus. *Mol. Biol. Evol.* 29 313–323. 10.1093/molbev/msr206 21878683

[B28] Okada K. Saito T. Nakagawa T. Kawamukai M. Kamiya Y. (2000). Five geranylgeranyl diphosphate synthases expressed in different organs are localized into three subcellular compartments in *Arabidopsis*. *Plant Physiol.* 122 1045–1056. 10.1104/pp.122.4.1045 10759500 PMC58939

[B29] Prchalová D. Buèek A. Brabcová J. Žáèek P. Kindl J. Valterová I. (2016). Regulation of isoprenoid pheromone biosynthesis in bumblebee males. *Chembiochem* 17 260–267. 10.1002/cbic.201500415 26632352

[B30] Quondam M. Barbato C. Pickford A. Helmercitterich M. Macino G. (1997). Homology modeling of *Neurospora crassa* geranylgeranyl pyrophosphate synthase: structural interpretation of mutant phenotypes. *Protein Eng. Des. Sel.* 10 1047–1055. 10.1093/protein/10.9.1047 9464568

[B31] Ruiz-Sola M. A. Coman D. Beck G. Barji M. V. Colinas M. Graf A. (2016). *Arabidopsis* geranylgeranyl diphosphate synthase 11 is a hub isozyme required for the production of most photosynthesis-related isoprenoids. *New Phytol.* 209 252–264. 10.1111/nph.13580 26224411

[B32] Sagami H. Korenaga T. Ogura K. (1993). Geranylgeranyl diphosphate synthase catalyzing the single condensation between isopentenyl diphosphate and farnesyl diphosphate. *J Biochem.* 114 118–121. 10.1093/oxfordjournals.jbchem.a124125 8407863

[B33] Shimizu I. Kitabatake S. Kato M. (1981). Effect of carotenoid deficiency on photosensitivities in the silkworm, *Bombyx mori*. *J. Insect Physiol.* 27 593–599. 10.1016/0022-1910(81)90106-2

[B34] Singkaravanit S. Kinoshita H. Ihara F. Nihira T. (2010). Geranylgeranyl diphosphate synthase genes in entomopathogenic fungi. *Appl. Microbiol. Biotechnol.* 85 1463–1472. 10.1007/s00253-009-2171-9 19690851

[B35] Sloan D. B. Moran N. A. (2012). Endosymbiotic bacteria as a source of carotenoids in whiteflies. *Biol. Lett.* 8 986–989. 10.1098/rsbl.2012.0664 22977066 PMC3497135

[B36] Sun Z. J. Li Z. X. (2018). The terpenoid backbone biosynthesis pathway directly affects the biosynthesis of alarm pheromone in the aphid. *Insect Mol. Biol.* 27 824–834. 10.1111/imb.12521 30039630

[B37] Swanson K. M. Hohl R. J. (2006). Anti-cancer therapy: targeting the mevalonate pathway. *Curr. Cancer Drug Targets* 6 15–37. 10.2174/156800906775471743 16475974

[B38] Tamura K. Peterson D. Peterson N. Stecher G. Nei M. Kumar S. (2011). MEGA5: molecular evolutionary genetics analysis using maximum likelihood, evolutionary distance, and maximum parsimony methods. *Mol. Biol. Evol.* 28 2731–2739. 10.1093/molbev/msr121 21546353 PMC3203626

[B39] Tsuchida T. (2016). Molecular basis and ecological relevance of aphid body colors. *Curr. Opin. Insect Sci.* 17 74–80. 10.1016/j.cois.2016.07.005 27720077

[B40] Tsuchida T. Koga R. Horikawa M. Tsunoda T. Maoka T. Matsumoto S. (2010). Symbiotic bacterium modifies aphid body color. *Science* 330 1102–1104. 10.1126/science.1195463 21097935

[B41] Valmalette J. C. Dombrovsky A. Brat P. Mertz C. Capovilla M. Robichon A. (2012). Light-induced electron transfer and ATP synthesis in a carotene synthesizing insect. *Sci. Rep.* 2:sre00579. 10.1038/srep00579 22900140 PMC3420219

[B42] Varney M. L. Goetz D. B. Wiemer D. F. Holstein S. A. (2018). Isoprenoid amide bisphosphonates as a novel class of geranylgeranyl diphosphate synthase inhibitors. *Blood* 132:4679 10.1182/blood-2018-99-112965

[B43] Waller D. D. Park J. Tsantrizos Y. S. (2019). Inhibition of farnesyl pyrophosphate (FPP) and/or geranylgeranyl pyrophosphate (GGPP) biosynthesis and its implication in the treatment of cancers. *Crit. Rev. Biochem. Mol.* 54 41–60. 10.1080/10409238.2019.1568964 30773935

[B44] Waterhouse A. M. Procter J. B. Martin D. M. A. Clamp M. Barton G. J. (2009). Jalview Version 2—a multiple sequence alignment editor and analysis workbench. *Bioinformatics* 25 1189–1191. 10.1093/bioinformatics/btp033 19151095 PMC2672624

[B45] Wiemer A. J. Wiemer D. F. Hohl R. J. (2011). Geranylgeranyl diphosphate synthase: an emerging therapeutic target. *Clin. Pharmacol. Ther.* 90 804–812. 10.1038/clpt.2011.215 22048229

[B46] Yang C. Pan H. Liu Y. Zhou X. (2014). Selection of reference genes for expression analysis using quantitative real-time PCR in the pea aphid, *Acyrthosiphon pisum* (Harris) (Hemiptera, Aphidiae). *PLoS One* 9:e110454. 10.1371/journal.pone.0110454 25423476 PMC4244036

[B47] Yang L. E. Huang X. Q. Lu Q. Q. Zhu J. Y. Lu S. (2016). Cloning and characterization of the geranylgeranyl diphosphate synthase (GGPS) responsible for carotenoid biosynthesis in *Pyropia umbilicalis*. *J. Appl. Phycol.* 28 671–678. 10.1007/s10811-015-0593-6

[B48] Ye C. An X. Jiang Y. D. Ding B. Y. Shang F. Christiaens O. (2019). Induction of RNAi core machinery’s gene expression by exogenous dsRNA and the effects of pre-exposure to dsRNA on the gene silencing efficiency in the pea aphid (*Acyrthosiphon pisum*). *Front. Physiol.* 9:1906. 10.3389/fphys.2018.01906 30687121 PMC6333656

[B49] Yi P. Han Z. Li X. Olson E. N. (2006). The mevalonate pathway controls heart formation in drosophila by isoprenylation of Gγ1. *Science* 313 1301–1303. 10.1126/science.1127704 16857902

[B50] Zhang H. Li Z. X. (2014). A type-III insect geranylgeranyl diphosphate synthase with a novel catalytic property. *Protein Pept. Lett.* 21 615–623. 10.2174/0929866521666140214123942 24527738

[B51] Zhang J. C. Tao N. G. Xu Q. Zhou W. J. Cao H. B. Xu J. (2009). Functional characterization of citrus PSY gene in Hongkong kumquat (*Fortunella hindsii* swingle). *Plant Cell Rep.* 28 1737–1746. 10.1007/s00299-009-0774-3 19813015

[B52] Zhang Y. Wang X. X. Zhu J. Y. Zhang Z. F. Tian H. G. Liu T. X. (2016). Strategies used by two apterous strains of the pea aphid *Acyrthosiphon pisum* for passive disperal. *Biol. Open* 5 1535–1544. 10.1242/bio.018903 27628035 PMC5087678

[B53] Zhou F. Wang C. Y. Gutensohn M. Jiang L. Zhang P. Zhang D. (2017). A recruiting protein of geranylgeranyl diphosphate synthase controls metabolic flux toward chlorphyll biosynthesis in rice. *Proc. Natl. Acad. Sci. U.S.A.* 114 6866–6871. 28607067 10.1073/pnas.1705689114 PMC5495272

