# Supplementary figures and images for: Characterization of the Geranylgeranyl Diphosphate Synthase Gene in Acyrthosiphon pisum (Hemiptera: Aphididae) and Its Association With Carotenoid Biosynthesis
Source: Front Physiol. 2019 Nov 12;10:1398. doi: 10.3389/fphys.2019.01398 (PMC6861191; doi:10.3389/fphys.2019.01398)

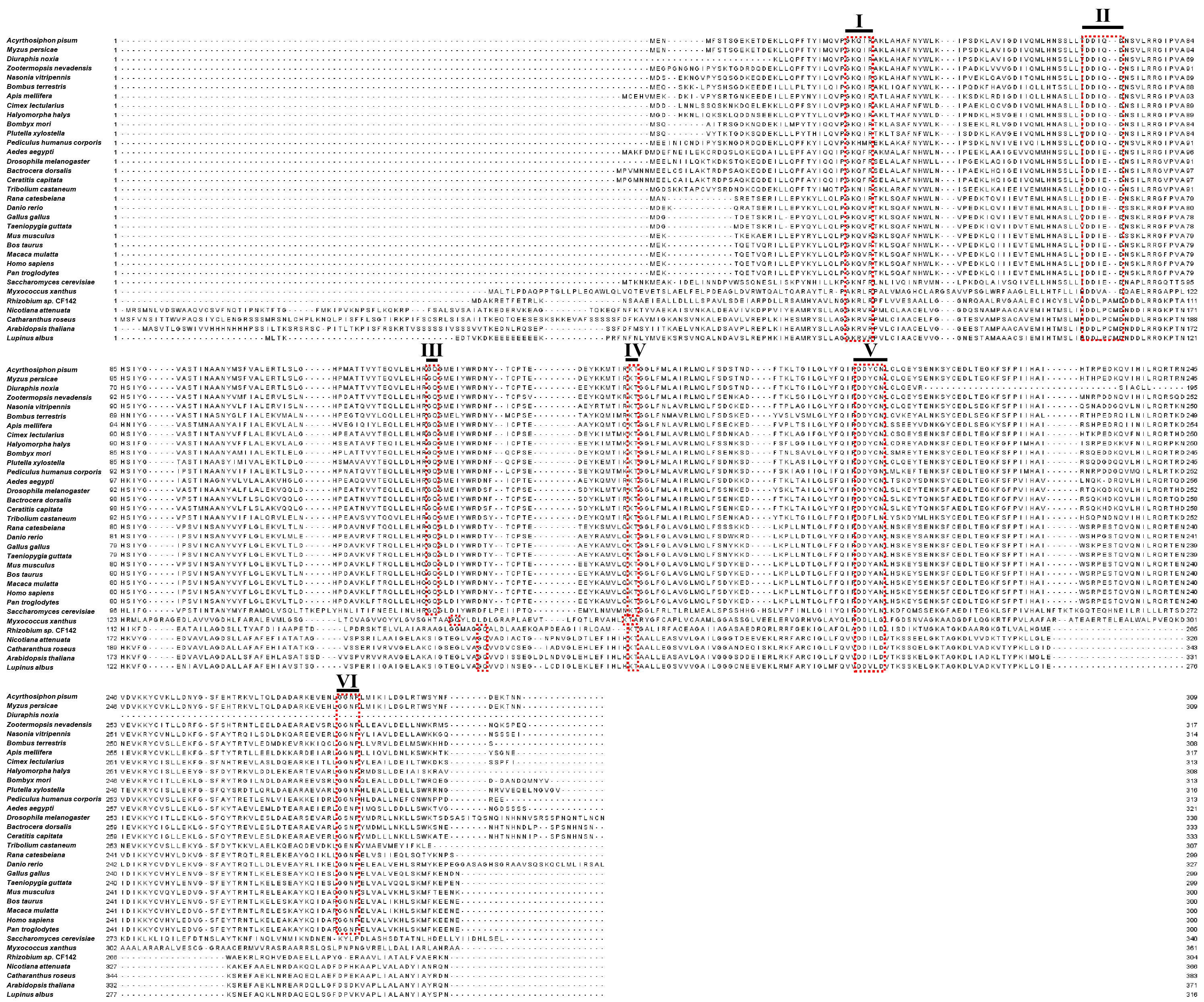

Supplement: FIGURE S6 — Alignment of the amino acid sequence of Acyrthosiphon pisum GGPPS with GGPPS from other species. Conserved motifs are indicated by the red box with dotted lines. [file Image_6.TIF]
