# Supplementary material for: Characterization of the Geranylgeranyl Diphosphate Synthase Gene in Acyrthosiphon pisum (Hemiptera: Aphididae) and Its Association With Carotenoid Biosynthesis
Source: Front Physiol. 2019 Nov 12;10:1398. doi: 10.3389/fphys.2019.01398 (PMC6861191; doi:10.3389/fphys.2019.01398)
Supplement: TABLE S1 — Primer sequences used for sequence confirmation, quantitative real-time PCR (qRT-PCR) and double stranded RNA (dsRNA) synthesis. [file Table_1.DOC]

**Table S1. Primer sequences used for sequence confirmation, quantitative real-time PCR (qRT-PCR) and double stranded RNA (dsRNA) synthesis.**

| Experiments | Primer names and sequence (5’ to 3’) | Amplification efficiency |
| --- | --- | --- |
| Full length confirmation | *GGPPS*-F: ATGGAAAATATGTTTAGTAC  *GGPPS*-R: TAATTGTTTGTCTTTTCATC  Q-*GGPPS*-F: ACCCGTGGCTCATTCAATCT  Q-*GGPPS*-R: TTCCATTCCTTGACCTCGGT | ——  101.0% |
| RT-qPCR |  |  |
|  | Q-*CscA*-F: TTCCTATGGTACGGTGCTGG  Q-*CscA*-R: CGCCCAAGACGATTATCACG  Q-*CscB*-F: CTTGTGAACATTGCCCGTGA  Q-*CscB*-R: GAACCGAACCTCTTGTGACG  Q-*CscC*-F: GCAAACCGTTCTCACTTCGT  Q-*CscC*-R: CGCCCAACACAGTATGGATC | 96.6% |
|  |  | 103.3% |
|  |  | 98.8% |
|  | Q-*CdeA*-F: AGCCAGAGAACACGTGATCA  Q-*CdeA*-R: GGCTGTACTGATGCACCAAC | 98.7% |
|  | Q-*CdeB*-F: TGCGTACAACAAACTTTTGCCA  Q-*CdeB*-R: AAACGATGGATCATCGGGCA | 109.2% |
|  | Q-*CdeC*-F: TCATCATCGGAGCTGGAGTT  Q-*CdeC*-R: TGAAGGGCCTTGATCAAATCG | 104.1% |
|  | Q-*CdeD*-F: ATACGCCAATAAGCTCGGGA  Q-*CdeD*-R: ATGGTTCATCGGGCAAAGTG | 101.0% |
|  | Q-*EF1α*-F: CTGTGCTTATTGTCGCTGCT  Q-*EF1α*-R: TCGCTGTATGGTGGTTCAGT | 108.9% |
|  | Q-*RPS20*-F: AAGTGTGTGCTCCGAGATGA  Q-*RPS20*-R: CAGCAATGACACCGGGTTC | 98.0% |
| dsRNA synthesis | ds*GGPPS*-F: taatacgactcactatagggATATGTTTAGTACGTCCGG | —— |
|  | ds*GGPPS*-R: taatacgactcactatagggGTGTATACAGTGGTAGCCA |  |
|  | ds*GFP*-F: taatacgactcactatagggCAGTTCTTGTTGAATTAGATG | —— |
|  | ds*GFP*-R: taatacgactcactatagggTTTGGTTTGTCTCCCATGATG |  |

*Csc*, carotenoid synthase/ cyclase, *CscA* (Accession: XP_001943170), *CscB* (Accession: XP_003241668.1) and *CscC* (Accession: XP_001950787); *Cde*, carotenoid desaturase, *CdeA* (Accession: XP_001943225), *CdeB* (Accession: NP_001171302.1), *CdeC* (Accession: XP_001946689) and *CdeD* (Accession: XP_001950764); *EF1α*, *elongation factor 1α*; *RPS20*, *ribosomal protein S20-like*; dsRNA, double-stranded RNA; GFP, Green fluorescent protein; F, forward; R, reverse. The lowercase letters represent t the T7 promoter sequences for efficient in vitro transcription in dsRNA synthesis.
